# Factors Affecting Portal Usage Among Chronically Ill Patients During the COVID-19 Pandemic in the Netherlands: Cross-sectional Study

**DOI:** 10.2196/26003

**Published:** 2021-07-19

**Authors:** Qingxia Kong, Danique Riedewald, Marjan Askari

**Affiliations:** 1 Rotterdam School of Management Erasmus University Rotterdam Netherlands; 2 Erasmus School of Health Policy & Management Erasmus University Rotterdam Netherlands

**Keywords:** COVID-19, pandemic, digital technology, eHealth, patient portals, chronically ill patients, portal responsiveness, portal awareness

## Abstract

**Background:**

The COVID-19 pandemic has impacted the capacity of the regular health care system, which is reflected in limited access to nonurgent care for patients who are chronically ill in the Dutch health care system. Nevertheless, many of them still depend on health care assistance to manage their illnesses. Patient portals are used to provide continued health care (remotely) and offer self-management tools during COVID-19 and potentially after. However, little is known about the factors influencing portal use and users’ satisfaction among patients who are chronically ill during the COVID-19 pandemic.

**Objective:**

This study aims to examine predictors of patient portal use among patients who are chronically ill, the willingness to recommend the portal to others, and the likelihood of future use among portal nonusers.

**Methods:**

An online self-administered questionnaire was distributed among patients who are chronically ill via social media in May 2020. The questionnaire consisted of four parts: (1) demographics including age and hours of daily internet use; (2) physical health status including COVID-19 infection, perceived level of control, and hospital visits; (3) mental health status including depression and life satisfaction; and (4) portal use including response waiting time and awareness. Descriptive, correlation, univariate, and multivariate analyses were conducted to identify factors that affect portal use, users’ willingness to recommend, and nonusers’ likelihood of future portal use.

**Results:**

A total of 652 patients responded, and 461 valid questionnaires were included. Among the 461 patients, 67% (n=307) were identified as patient portal users. Of the nonusers, 55% (85/154) reported not being aware of the existence of a patient portal at their hospital. Significant predictors of portal use include level of control (*P*=.04), hospital visit time (*P*=.03), depression scale (*P*=.03), and status of life satisfaction (*P*=.02). Among portal users, waiting time to get a response via the portal (*P*<.001) and maximum acceptable waiting time (*P*<.001) were the strongest predictors for willingness to recommend the portal; among nonusers, the model predicted that those who were not aware of patient portals (*P*<.001) and were willing to wait moderately long (*P*<.001) were most likely to use the portal in the future.

**Conclusions:**

This study provides insights into factors that influence portal use and willingness to recommend, based on which health care providers can improve the adoption of patient portals and their services. It suggests that health care providers should leverage efficient operations management to improve responsiveness and reduce waiting time to enhance user satisfaction and willingness to recommend use. Health care organizations need to increase portal awareness among nonusers and train their patients to increase both use and longer adoption of patient portals. Factors including depression and life satisfaction can influence portal use; therefore, future studies on determinants of portal use and nonuse in this specific population are needed.

## Introduction

On January 30, 2020, COVID-19 was officially declared a pandemic by the World Health Organization [1]. As a result of the virus outbreak, the Netherlands, along with other countries, announced a lockdown. This lockdown, called an *intelligent lockdown,* entailed that people were encouraged (not forced) to stay inside as much as possible, social gatherings with more than three people were prohibited, and many (nonessential) businesses were temporarily closed [2]. The primary purpose of this *intelligent lockdown* was to prevent peak loads of patients requiring intensive care [3]. The pandemic’s consequences were a massive burden on the Dutch health care system, particularly in the initial period of the outbreak (March 2020). Intensive care units were struggling with allocating their capacity, causing patients to be distributed over various hospitals throughout the Netherlands [4]. Meanwhile, regular health care was disrupted due to the COVID-19 outbreak. To alleviate the pressure of health care professionals and to prevent them and nonurgent patients from infecting each other [5], nonurgent patients’ appointments were canceled, postponed, or moved online [6,7]. Several experts and health care professionals subsequently proposed eHealth as a solution for the continuation of care for patients who are chronically ill [8,9].

According to a study supported by the Dutch government, approximately 5.3 million Dutch patients have one or more chronic illnesses. This number is expected to rise to 7 million by 2030 [10]. In Europe, about 70% to 80% of the total health care budget is spent on treating and preventing chronic disease [11], which indicates that chronic illness is a common issue with an enormous financial burden. Two critical elements of chronic care are frequent contact with their care providers and self-management (eg, adapting to their condition and learning to deal with their disease) [12]. Therefore, some still rely on regular nonurgent health care and need assistance during the COVID-19 pandemic to keep their illness under control. Limited access to care, in addition to the fear of contracting the virus, getting sick, or even passing away, could potentially lead to diminished (perceived) physical and mental health outcomes for this group of patients [13,14]. Indeed, previous studies have shown that people with chronic diseases are more prone to anxiety and depression than those without [15,16]. Although care for patients with COVID-19 requires the most attention during this crisis, it is crucial to continue to provide patients who are chronically ill the care they need, including offering self-management tools, monitoring, controlling, and disease treatment. It will ultimately reduce the risk of emergency care and hospital admission, and prevent long-term complications in these patients [17].

During the COVID-19 pandemic, eHealth has been suggested as a valuable solution to provide care to patients who are chronically ill, enabling self-management of chronic conditions and providing care remotely and safely [18,19]. The Dutch government has compiled a subsidy program (VIPP) to accelerate the implementation of eHealth solutions in specialized medical care organizations throughout the country [20]. By 2019, 60 out of 73 Dutch hospitals offered an eHealth solution [21]. The solution is essentially a platform called a *patient portal*. In these patient portals, patients are, among other things, able to investigate their electronic health records, directly message their health care practitioner, and view their laboratory results along with personal details. Each hospital was allowed to decide on the functionalities implemented in its patient portal. Despite the VIPP implementation, several reports provide evidence on the lack of patient engagement, reflected in a large portion of nonusers [22].

Therefore, it is crucial to understand which factors influence portal use for patients who are chronically ill, users’ satisfaction, and nonusers’ likelihood of future use to promote the adoption of patient portals and retain current users. A retrospective cohort study among the adult patient population found that those who are younger, are White, have commercial insurance, and have higher annual income are more likely to be portal users [23]. Another cross-sectional survey also found that age and income are significant predictors of portal adoption [24]. A cross-sectional survey among adult patients of a university hospital revealed that being chronically ill and having higher eHealth literacy were the best predictors for portal use [22]. However, it remains unknown which factors influence portal use among the patient group of interest—patients who are chronically ill—and, in particular, whether and how perceived physical and mental health conditions play a role during the COVID-19 pandemic. Besides, several papers published after the outbreak of COVID-19 studied patient satisfaction on patient portals or telehealth [25]. However, those studies are mainly descriptive (ie, they survey how many patients are satisfied with their experience rather than predicting or investigating the causal relationship). This study contributes to understanding which factors predicted portal use, portal users’ satisfaction, and portal nonusers’ likelihood of future adoption among patients who are chronically ill during the COVID-19 pandemic.

The research questions of this study are what factors affect patient portal use among patients who are chronically ill during the COVID-19 pandemic in the Netherlands and what factors affect portal users’ willingness to recommend and nonusers’ likelihood of using patient portals during the COVID-19 pandemic in the Netherlands?

## Methods

### Study Design and Procedure

A cross-sectional study was designed using an online self-administered questionnaire (the survey is available upon request). The survey was written in English and then translated into Dutch and verified by a person proficient in Dutch. The questionnaire was distributed throughout several Facebook groups aimed at (peer) support and providing information for patients who are chronically ill in May 2020.

After displaying the introduction of the questionnaire, informed consent was obtained electronically, before actual enrollment. It was explicitly stated that participation was voluntary, and participants could withdraw at any time without any consequences. Moreover, complete anonymity of the response was ensured.

During the period of data collection, the number of Dutch people who tested positive for COVID-19 exceeded 40,000, over 11,000 people were hospitalized, and almost 6000 deaths related to COVID-19 had been reported in a population of 17 million inhabitants [26]. When distributing the questionnaire, the national *intelligent lockdown* had been active for approximately 1.5 months. As compensation for the time spent on the survey, online gift codes were distributed through a raffle. To ensure good quality responses, some survey items were programmed to be restricted in range so that incorrect inputs were not allowed.

This study followed the Institutional Review Board (IRB) for the Protection of Human Subjects Guidelines. All procedures in this study were approved by the IRB (2020/04/24-61392qko) prior to its initiation.

### Participants

Since the study focused on patient portals as implemented by Dutch hospitals, the targeted population for our study was patients who are chronically ill and residing in the Netherlands. Inclusion criteria were as follows: patients aged between 18 and 65 years, having at least one chronic illness, and having spent more than 2 minutes completing the questionnaire. Questionnaires that were not completed were removed from the final data set.

### Measures

#### Demographic Characteristics

Demographic variables included participant’s gender (male, female, or other), age, highest educational level completed, main occupation, yearly income, chronic illness or illnesses, hours of daily internet use, and portal use (yes or no).

#### Physical Health Status and Hospital Visits

Physical health status was assessed using four categories: (1) COVID-19 status, (2) level of control over chronic illness, (3) lifestyle and exercise, and (4) perceived health. COVID-19 status was assessed by inquiring about the prevalence of any COVID-19 symptoms over the last 2 weeks (yes, no, or unsure), COVID-19 testing (yes or no), and COVID-19 infection (yes, no, or unsure). Level of control over chronic illness was assessed using a single 5-point item, asking people to rate their current level of control over their chronic illness (totally in control to not at all in control). Lifestyle was assessed using a common measure of lifestyle and activity [27,28]. Exercise was measured by the frequency of exercise in the last 2 weeks. Perceived health was measured using the Self-Rated Health measure, a widely used, single-item measure of self-perceived health status [29]. The item consisted of one question (“In general, would you say your health is:”) with five answer options between 1 (excellent) and 5 (poor) [30]. Finally, patients’ frequency of hospital visits and their durations were also measured.

#### Mental Health

Mental health was assessed using questions about both depression severity and life satisfaction. Depression severity was measured using the Patient Health Questionnaire 9 assessment scale, which is generally used to aid clinicians in diagnosing, monitoring, and treating depressive symptoms and their severity [31]. Patients score nine different items on a scale of 0 (not at all) to 3 (nearly every day). The scores are then summed up to achieve a final score, which can be assessed by the clinician or researcher over a few cut-off categories. The categories are 0 to 4, 5 to 9, 10 to 14, 15 to 19, and 20 to 27, in sequence of increasing depression severity [32]. General well-being was assessed by examining participants’ satisfaction with life, using the Satisfaction with Life Scale [33]. The tool allows participants to self-report their opinions regarding the satisfaction they experience with their own lives. The scale contains five items, and participants report their answers over a 7-point Likert scale (1, strongly disagree, to 7, strongly agree). After the assessment, scores are summed up to arrive at a final score. The outcomes are categorized as 5 to 9 (extremely dissatisfied), 10 to 14 (dissatisfied), 15 to 19 (slightly dissatisfied), 20 (neutral), 21 to 25 (slightly satisfied), 26 to 30 (satisfied), and 31 to 35 (extremely satisfied).

#### Patient Portal Use

Participants were identified as portal users if they selected *yes* to the question “Have you ever used a patient portal?” Among portal users, their portal use was assessed by frequency of use, time duration of use, and waiting time. The time of use measures the average duration each time a patient uses a portal, and frequency of use indicates how often a patient uses a portal. They are two dimensions of patient engagement with the portal. Furthermore, the waiting time from sending a request until receiving a reply was recorded. For patient portal users (ie, people who have used a portal before), their usual and maximum acceptable waiting time was asked. In contrast, for nonusers, only the maximum acceptable waiting time was recorded. Lastly, portal users’ willingness to recommend the portal to others—a strong indicator of customer loyalty and predictor for growth [34]—was measured by a single question: “Would you recommend the patient portal to others?” Portal nonusers were asked to input a percentage value (from 0 to 100) to answer the question “What is your likelihood of using such a portal?” to measure their likelihood of future portal use.

### Data Analysis

Descriptive analysis was performed to gain insight into the patient population, portal users, and nonusers regarding their demographics, physical health status, hospital visits, and mental health status. Additionally, chi-square tests (for categorical variables) and Welch *t* tests (for numerical variables) were performed to compare the characteristics between portal users and nonusers. Next, univariate analyses were performed for the dependent variable (portal use) to detect its possible predictors. Variables with *P*<.20 in the univariate regression were consequently included in the multiple regression analysis after considering the correlation between variables (using statistical analysis and expert opinion). A stepwise backward elimination was then applied to reduce the number of independent variables and obtain the final multiple regression model. This approach allowed thorough exploration and testing of possible predictors to arrive at a final model [35]. Similar procedures were applied to the other two dependent variables (willingness to recommend among portal users and likelihood of use among portal nonusers) to get the final multiple regression models. All analyses were performed using RStudio (version 4.0.2; RStudio, PBC).

## Results

### Descriptive Analysis

A total of 652 respondents started the questionnaire, whereby 461 respondents completed it successfully. Only completed questionnaires were used in the final data set for analysis. Of all the participants, 307 (66.6%) reported to have used a patient portal, and 154 (33.4%) reported that they had not used a patient portal until the moment the survey was conducted.

Demographics of all participants, portal users, and nonusers are displayed in Table 1. From our sample (N=461), 94 (20.4%) were male, 365 (79.2%) were female, and 2 (0.4%) individuals identified as other. The mean age of the sample was 42.9 (SD 13.0) years. The number of participants that reported having a single chronic disease was 302 (65.5%), and 159 (34.5%) reported having multiple chronic diseases. Significant differences were noted in the mean age (*P*=.008) and main occupations (*P*=.03) between portal users and nonusers.

Table 2 displays the physical health status, hospital visits, and mental health status of all participants, portal users, and nonusers. The majority of the 461 patients reported to have their illness “a little bit” (n=113, 24.5%) to “moderately in control” (n=229, 49.7%), 66 (14.3%) reported to have total control, and 46 (10.0%) reported to have no control over their illness at all. Furthermore, few patients (n=30, 6.5%) reported that their perceived health was very good or excellent, while the majority reported good (n=127, 27.6%), fair (n=177, 38.4%), or poor (n=127, 27.6%) perceived health. Moreover, the majority of respondents reported spending 0.5 hours to 1 hour (n=151, 32.8%) and 1 hour to 2 hours (n=166, 36.0%) each time they visit a hospital (including travel time); 70 (15.2%) respondents spent less than half an hour, while only 19 (4.1%) spent more than 3 hours. Furthermore, only 18 (3.9%) participants reported having or having had COVID-19, 130 (28.2%) were uncertain, and 313 (67.9%) reported that they never had COVID-19. About mental health, most participants reported having no (n=172, 37.3%) to mild forms of (n=158, 34.3%) depression, while only 9.8% (n=45) reported having moderately severe or severe depression. Relative to life satisfaction, 146 (31.7%) and 117 (25.4%) of the participants were satisfied and slightly satisfied with their lives, respectively. Moreover, 27 (5.9%) and 28 (6.1%) were extremely satisfied and extremely dissatisfied with their lives, respectively. Among the measured characteristics, level of control (*P*=.005), average time of hospital visits (*P*=.04), depression (*P*=.02), and life satisfaction (*P*=.005) were significantly different between portal users and nonusers.

**Table 1:** Demographics of all the participants, portal nonusers, and users during the COVID-19 pandemic in the Dutch population of patients who are chronically ill.

Demographics		Total (N=461)	Nonusers (n=154)	Users (n=307)	*P* value
**Gender, n (%)**					.60
	Female	365 (79.2)	122 (79.2)	243 (79.2)	
	Male	94 (20.4)	32 (20.8)	62 (20.2)	
	Other	2 (0.4)	0 (0.0)	2 (0.7)	
Age (years), mean (SD)		42.9 (13.0)	45.1 (12.5)	41.8 (13.1)	.008
**Highest education, n (%)**					.06
	Primary school	13 (2.8)	8 (5.2)	5 (1.6)	
	Secondary/high school	82 (17.8)	29 (18.8)	53 (17.3)	
	MBO^a,b^ completed	201 (43.6)	73 (47.4)	128 (41.7)	
	HBO^c,d^ or university degree	155 (33.6)	41 (26.6)	114 (37.1)	
	Graduate degree	10 (2.2)	3 (1.9)	7 (2.3)	
**Main occupation, n (%)**					.03
	Self-employed	45 (9.8)	11 (7.1)	34 (11.1)	
	Employee	242 (52.5)	73 (47.4)	169 (55.0)	
	Student	35 (7.6)	9 (5.8)	26 (8.5)	
	Unemployed	117 (25.4)	50 (32.5)	67 (21.8)	
	Retired	22 (4.8)	11 (7.1)	11 (3.6)	
**Yearly income (€^e^), n (%)**					.29
	0-20,000	171 (37.1)	61 (39.6)	110 (35.8)	
	20,001-30,000	103 (22.3)	38 (24.7)	65 (21.2)	
	30,001-40,000	120 (26.0)	39 (25.3)	81 (26.4)	
	≥40,001	67 (14.5)	16 (10.4)	51 (16.6)	
**Chronic illness, n (%)**					.52
	Single chronic illness	302 (65.5)	104 (33.9)	198 (64.5)	
	Multiple chronic illnesses	159 (34.5)	50 (16.3)	109 (35.5)	
Daily internet use (hours), mean (SD)		5.7 (4.3)	5.4 (4.4)	5.8 (4.3)	.60

^a^MBO: Middelbaar beroepsonderwijs.

^b^English translation: secondary vocational education. It is oriented toward vocational training and is equivalent to a junior college education.

^c^HBO: Hoger beroepsonderwijs.

^d^English translation: higher professional education. It is oriented toward higher learning and professional training, and is the equivalent to a college education in the United States.

^e^A currency exchange rate of €1=US $1.18 is applicable.

**Table 2:** Physical health status, hospital visits, and mental health status of all the participants, portal nonusers, and users during the COVID-19 pandemic in the Dutch population of patients who are chronically ill.

Variables		Total (N=461), n (%)	Nonusers (n=154), n (%)	Users (n=307), n (%)	*P* value
**COVID-19 infection**					.72
	Yes	18 (3.9)	101 (65.6)	212 (69.1)	
	Not sure	130 (28.2)	46 (29.9)	84 (27.4)	
	No	313 (67.9)	7 (4.5)	11 (3.6)	
**Level of control**					.005
	Totally	66 (14.3)	28 (18.2)	38 (12.4)	
	Moderately	229 (49.7)	63 (40.9)	166 (54.1)	
	A little bit	113 (24.5)	41 (26.6)	72 (23.5)	
	Not at all	46 (10.0)	16 (10.4)	30 (9.8)	
	I don’t know	7 (1.5)	6 (3.9)	1 (0.3)	
**Perceived health (SRH^a^)**					.31
	Excellent	4 (0.9)	2 (1.3)	2 (0.7)	
	Very good	26 (5.6)	8 (5.2)	18 (5.9)	
	Good	127 (27.5)	36 (23.4)	91 (29.6)	
	Fair	177 (38.4)	57 (37.0)	120 (39.1)	
	Poor	127 (27.5)	51 (33.1)	76 (24.8)	
**Average time of hospital visit (hours)**					.04
	<0.5	70 (15.2)	32 (20.8)	38 12.4)	
	0.5-1	151 (32.8)	42 (27.3)	109 (35.5)	
	1-2	166 (36.0)	56 (36.4)	110 (35.8)	
	2-3	55 (11.9)	21 (13.6)	34 (11.1)	
	>3	19 (4.1)	3 (1.9)	16 (5.2)	
**Depression (PHQ-9^b^)**					.02
	None	172 (37.3)	56 (36.4)	116 (37.8)	
	Mild	158 (34.3)	49 (31.8)	109 (35.5)	
	Moderate	86 (18.7)	25 (16.2)	61 (19.9)	
	Moderately severe	33 (7.2)	15 (9.7)	18 (5.9)	
	Severe	12 (2.6)	9 (5.8)	3 (1.0)	
**Life satisfaction**					.005
	Extremely satisfied	27 (5.9)	6 (3.9)	21 (6.8)	
	Satisfied	146 (31.7)	44 (28.6)	102 (33.2)	
	Slightly satisfied	117 (25.4)	28 (18.2)	89 (29.0)	
	Neutral	21 (4.6)	10 (6.5)	11 (3.6)	
	Slightly dissatisfied	70 (15.2)	27 (17.5)	43 (14.0)	
	Dissatisfied	52 (11.3)	24 (15.6)	28 (9.1)	
	Extremely dissatisfied	28 (6.1)	15 (9.7)	13 (4.2)	

^a^SRH: Self-Rated Health.

^b^PHQ-9: Patient Health Questionnaire 9.

Table 3 reports the frequency of portal use before and after the lockdown. An increase in the frequency of portal use has been observed after the lockdown as compared to before, whereby the relative difference was 500%, 221.1%, and 8.3% in daily, weekly, and monthly use, respectively. After the lockdown, 67 (21.8%) reported daily to weekly use, and 106 (34.5%) have used the patient portal monthly. Among all the portal users, the most common use times were “5 minutes or less” (n=121, 39.4%) and “5-10 minute” (n=124, 40.4%), while 62 (20.2%) of them reported using the portals for more than 10 minutes.

In relation to the maximum acceptable waiting time, nonusers reported a lower maximum acceptable waiting time than users. Among the users, 78 (28.4%) reported a longer actual waiting time than they deem acceptable. Finally, among portal users, 257 (83.7%) would likely recommend portals to others, and among nonusers, the average likelihood of future use (ranging from 0% to 100%) was 53.6% (SD 33.3%).

**Table 3:** Descriptive of patient portal use before and after lockdown (n=307).^a^

Portal use	Frequency of use before lockdown, n	Frequency of use after initiation of lockdown, n	Relative difference (%)
(Almost) daily	1	6	500
Weekly	19	61	221.1
Monthly	56	106	89.3
Less than monthly	231	134	–42.0

^a^Relative comparison between periods translated according to relative frequency of use (period before the intelligent lockdown had a much larger timespan than the period after initiation of the intelligent lockdown and thus included portal nonusers).

### Multiple Regression Analysis

To investigate which combinations of the different predictors could best explain the variance in portal use versus nonuse and portal users’ willingness to recommend and portal nonusers’ likelihood of future use, three separate regression models were constructed after performing univariate regression analysis and considering possible correlations. In the first analysis (model 1), a logistic regression was performed to investigate the association between portal use and the included variables after the first steps, which were age, hospital visit time, level of control, depression, and life satisfaction. In the second analysis (model 2), a logistic regression was performed to study the relationship between portal users’ willingness to recommend and the variables average number of hours spent on the internet daily, the frequency of portal use after the COVID-19 lockdown in March 2020, waiting time for portal response, and maximum acceptable time to wait. In the third analysis (model 3), a multiple regression analysis was conducted between portal nonusers’ likelihood of use and age, income, maximum acceptable waiting time, and their awareness of patient portals’ existence as candidate variables. The results of the regression analysis are displayed in Table 4.

Regression results of model 1 showed that shorter hospital visit times (“less than half an hour”) predict less portal use (*β*=–.725; *P*=.03) compared to longer visit times. Compared to “totally under control,” moderate level of control predicts a higher chance (*β*=.629; *P*=.04*)* of portal use. Two mental health conditions were shown to significantly affect participants’ portal use. Participants with severe depression (*β*=–1.652; *P*=.03) and life dissatisfaction or extreme life dissatisfaction (*β*=–.844; *P*=.02) were found to be less likely to use patient portals. Furthermore, age demonstrates a small yet nonsignificant impact on portal use, whereby older age negatively affects portal use (*β*=–.015; *P*=.08).

Among portal users, the logistic regression results from model 2 showed that actual waiting time and maximum acceptable waiting time were the strongest predictors of users’ willingness to recommend. Participants whose average waiting time was between 1 to 2 days (*β*=–2.081; *P*<.001) or greater than 2 days (*β*=–1.784; *P*<.001) were less likely to recommend the portal system to others, compared to those who received responses via portal systems within 24 hours. Participants who reported a moderate maximum waiting time (1-2 days) were more likely to recommend portal systems (*β*=2.292; *P*<.001).

For portal nonusers (model 3), awareness of the portal existence was the strongest predictor besides maximum acceptable waiting time. Among nonusers, 85 (55.2%) reported being unaware of the existence of a patient portal at their hospital. Participants that were unaware of the existence of portal systems were 25.9% (*P*<.001) more likely to use portal systems, compared to those that already knew of their existence before the time of the survey. Participants who had a moderate maximum acceptable waiting time (12-24 hours) were 21.2% (*P*<.001) more likely to use portal systems in the future. Furthermore, middle income class participants (€30,001 [US $35,440.20] to €40,000 [US $47,252.00]) were 15.3% (*P*=.01) more likely to use portal systems compared to low income class participants (<€20,001 [US $23,627.20]), and older-aged participants also showed a slightly lower likelihood (*β*=–.003; *P*=.10*)* of use.

**Table 4:** Results of the multiple regression model, indicating the significant predictors.

Variables		Model 1 portal use (all participants)		Model 2 recommendation (portal users)		Model 3 likelihood of using (portal nonusers)	
		Estimates	*P* value	Estimates	*P* value	Estimates	*P* value
Intercept		1.513	.004	0.685	.10	0.579	<.001
Age		–0.015	.08	N/A^a^	N/A	–0.003	.10
**Income (€^b^; reference: 0-20,000)**							
	20,001-30,000	N/A	N/A	N/A	N/A	–0.00938	.88
	30,001-40,000	N/A	N/A	N/A	N/A	0.153	.01
	≥40,001	N/A	N/A	N/A	N/A	0.054	.51
Daily internet hours		N/A	N/A	0.093	.07	N/A	N/A
**Hospital visit time (hours; reference: 0.5-1)**							
	<0.5	–0.725	.03	N/A	N/A	N/A	N/A
	1-2	–0.246	.35	N/A	N/A	N/A	N/A
	2-3	–0.548	.12	N/A	N/A	N/A	N/A
	>3	0.613	.37	N/A	N/A	N/A	N/A
**Level of control (reference: totally)**							
	Moderately	0.629	.04	N/A	N/A	N/A	N/A
	Little bit	0.328	.36	N/A	N/A	N/A	N/A
	Not at all	0.823	.08	N/A	N/A	N/A	N/A
	I don’t know	–1.825	.11	N/A	N/A	N/A	N/A
**Depression scale (reference: mild)**							
	None	–0.147	.58	N/A	N/A	N/A	N/A
	Moderate	0.259	.43	N/A	N/A	N/A	N/A
	Moderately severe	–0.321	.45	N/A	N/A	N/A	N/A
	Severe	–1.652	.03	N/A	N/A	N/A	N/A
**Life satisfaction scale (reference: satisfied or more)**							
	Slightly satisfied	0.116	.69	N/A	N/A	N/A	N/A
	Neutral	–1.009	.05	N/A	N/A	N/A	N/A
	Slightly dissatisfied	–0.589	.08	N/A	N/A	N/A	N/A
	Dissatisfied or less	–0.844	.02	N/A	N/A	N/A	N/A
**Portal use COVID-19 (reference: daily)**							
	Weekly or more	N/A	N/A	1.269	.11	N/A	N/A
	3-5 times	N/A	N/A	2.050	.06.	N/A	N/A
	1-2 times	N/A	N/A	0.124	.74	N/A	N/A
**Waiting time (reference: less than 24 hours)**							
	1-2 days	N/A	N/A	–2.081	<.001	N/A	N/A
	>2 days	N/A	N/A	–1.784	<.001	N/A	N/A
	Never tried	N/A	N/A	–0.911	.16	N/A	N/A
	No possibility	N/A	N/A	–0.681	.35	N/A	N/A
**Maximum acceptable waiting time (reference: <12 hours)**							
	12-24 hours	N/A	N/A	1.187	.006	0.212	<.001
	1-2 days	N/A	N/A	2.292	<.001	0.192	.006
	>2 days	N/A	N/A	1.502	.03	0.181	.05
Awareness (reference: yes)		N/A	N/A	N/A	N/A	0.259	<.001
Participants, n		461	N/A	307	N/A	154	N/A
Akaike information criterion		570.87	N/A	246.96	N/A	N/A	N/A
R^2^		N/A	N/A	N/A	N/A	0.3554	N/A
Adjusted R^2^		N/A	N/A	N/A	N/A	0.2904	N/A

^a^N/A: not applicable.

^b^A currency exchange rate of €1=US $1.18 is applicable.

## Discussion

### Main Findings and Comparison With Other Studies

Although the societal and health impacts of the COVID-19 pandemic have been present for nearly a year, there is no evidence on factors that affect patient portal adoption among patients who are chronically ill during the COVID-19 pandemic. Moreover, little research has been done on what influences users’ willingness to recommend and nonusers’ likelihood of using patient portals during the COVID-19 pandemic. Our findings portray some interesting insights for portal service providers and health care professionals.

In the participant population under study, we found that almost 67% (307/461) of participants were portal users, which is much higher than for general patient populations reported [36-38]. For example, Griffin et al [36] found in their study that 83.4% of patients were nonusers of the UNC Chart patient portal among a general patient population. It could be attributed to the difference in the study population and the impact of the COVID-19 pandemic. Ancker et al [39] found that patients with chronic illness were more likely to use a patient portal. Table 3 shows that both the number of portal users and frequency of use have increased significantly after the lockdown initiation in spring 2020. We found that participants whose level of control was moderate had a higher likelihood of using portal systems than participants with total control. This may be attributed to participants’ perception whereby they deem a portal as unnecessary when their health is well managed and under control.

Besides, we found that participants with shorter visit times to a hospital have a reduced likelihood of portal use compared to those with longer visit times. As reported in many other studies [40-42], savings on travel time and cost are among the major benefits of eHealth. This result suggests that the convenience of physical visits most likely reduces remote visits using patient portals. Furthermore, participants with severe depression and lower life satisfaction tend to use patient portals less. Mental health problems likely deter patients from using portal systems. This result coincides with the observation that patients with chronic anxiety and depression are less likely to be intense eHealth users [37]. Future studies should focus on determinates of portal use and nonuse in this specific population.

Our results show that older age may negatively affect portal use. It is in line with a recent study in the older population on the intention to use medical applications. Feelings of having control, service availability, perceived ease of use and usefulness, and attitude toward the medical application affect the intention to use in older adults, which may be attributed to anxiety triggered by technology use, lack of privacy, or trust [43]. Another study also argues that this is probably because older people often lack the infrastructure, knowledge, and skills needed to use eHealth programs [44]. Future studies are required to investigate determinants of portal use and nonuse in the older adult population.

Willingness to recommend patient portal systems was also investigated. No less than 83.7% reported willingness to recommend the portal to family and friends, which suggests that most users were satisfied and loyal with their hospital’s patient portal system [34]. The average waiting time to receive a response was a strong predictor for users’ positive experience using portal systems. Approximately 29% of patients reported receiving responses within 2 hours of a request, which is considered rapid. Numerous studies in the appointment scheduling area have shown the importance of managing waiting time in health care management [45-47]. Marketing research has shown that waiting time is a crucial determinant of customer satisfaction and loyalty [48]. Nonusers seem to expect faster response rates from patient portals than users. Palawatta [49] demonstrated that if nonusers perceive the response rate is longer than their perceived acceptable waiting time, they will feel less satisfied and, therefore, less inclined to try the portal system. Users, if they experience disconfirmation in waiting time and maximum acceptable waiting time, are likely to be less satisfied and therefore less likely to remain committed to using the portal system. These are essential insights for health care practitioners and managers to leverage operational efficiencies such as appointment scheduling and resource allocation.

Among the nonuser group, the majority (85/154, 55%) reported not being aware of a patient portal system at their hospital. Awareness of portal systems was found to be the largest predictor for future use in our study. It seems that many patients do not use portals partly due to unawareness of their existence. This result is in line with Griffin et al [36], who found that patients often did not use patient portals simply because they were unaware of their existence. This result suggests that enhancing the awareness of portal systems is the first step for health care organizations to take to increase portal use.

### Limitations and Future Research

There are some limitations bound to this study. First, the survey is cross-sectional, making it impossible to make causal claims, limiting the study to predictions only. Furthermore, the study relies on self-reported data on portal use. This is because when the study was implemented, we did not have access to the actual use data, such as log data of portal users. Besides, this survey focused on both portal users and nonusers to study factors that influence portal use and future use of nonusers. This, for example, cannot be replicated by merely approaching the actual users. However, it would be more insightful to use real use data (eg, log data retrieved from the portal) to establish the length and frequency of use. We suggest this as a future study when access to portal data is possible.

Second, the study invited participants via social media (Facebook peer support group) to complete the self-administered questionnaire. On one hand, sampling from Facebook support groups has apparent benefits, such as convenience and its focus on the targeted population. On the other hand, it also has a few known biases [50,51]. For example, Facebook excludes people who have a lower eHealth literacy, one important predictor of portal use among adult patients [22]. Besides, not everybody uses Facebook, especially older people. Although this problem is partly compensated by focusing on the age group 18 to 65 years, our results might overestimate the proportion of portal users among the total population. This partly explains why the ratio of portal users is higher than reported in many other studies. Little is known about the characteristics of people who do not use technology and why they do not use the portal. We suggest that future studies should focus on older people and people with less eHealth literacy.

Moreover, more females than males participated in this study. According to Smith [52], females are more likely to respond to (online) surveys than males. The authors proposed different reasons that could be grounded to this observation, including behavioral differences between males and females in relation to the internet or inherent internal feelings. Another study [53] found similar results (70% female response).

Finally, it is important to see which functionalities users use and the respective frequency to understand the perceived value of these functionalities to patients. This will potentially improve the frequency of use and tailoring portal systems according to the needs of patient. Future research could build on our results, aimed at further investigation of the use dimension of patient portals.

### Conclusion

Individuals that have spent less time on physical hospital visits, whose health is moderately under control or with severe depression or lower life satisfaction are less likely to use patient portal systems. Among users, short waiting time was the most important predictor for satisfaction of portal use, and among nonusers, awareness was the most important predictor of future portal system use. These findings provide insights for health care providers on how to promote patient portal use and improve user satisfaction.

## Abbreviations

IRB: Institutional Review Board
